# Susceptibility of *Lactobacillaceae* Strains to Aminoglycoside Antibiotics in the Light of EFSA Guidelines

**DOI:** 10.3390/life15050732

**Published:** 2025-04-30

**Authors:** Marta Dec, Klaudia Herman-Ostrzyżek, Aldert Zomer, Renata Urban-Chmiel

**Affiliations:** 1 Department of Veterinary Prevention and Avian Diseases, University of Life Sciences in Lublin, 20-612 Lublin, Poland; klaudia.herman@up.lublin.pl (K.H.-O.); renata.urban@up.lublin.pl (R.U.-C.); 2 Division of Infectious Diseases and Immunology, Faculty of Veterinary Medicine, Utrecht University, 3584 CL Utrecht, The Netherlands; A.L.Zomer@uu.nl; 3 WHO Collaborating Centre for Reference and Research on Campylobacter and Antimicrobial Resistance from a One Health Perspective/WOAH Reference Laboratory for Campylobacteriosis, 3584 CL Utrecht, The Netherlands

**Keywords:** *Lactobacillaceae*, probiotics, EFSA, feed additives, susceptibility testing, aminoglycosides

## Abstract

*Lactobacillaceae* is a large family of bacteria from which probiotic strains often originate. Microorganisms used as feed additives in the EU must meet a number of formal criteria, some of which concern antimicrobial susceptibility. In this study, we determined the susceptibility of 19 reference strains and 121 wild-type strains of *Lactobacillaceae* to aminoglycoside antibiotics using the broth microdilution method based on the ISO 10932:2010/IDF 223:2010 standard. Strains were categorized as resistant or susceptible according to European Food Safety Authority (EFSA) guidelines. Resistance genes were detected by whole genome sequence (WGS) analysis or by PCR. The MICs read after 48 h of incubation showed that 36.8% of reference strains were resistant to kanamycin, 26.3% to streptomycin, and 5.3% to gentamicin, with no aminoglycoside resistance genes detected in any genome. As many as 93.2% of field isolates of *Ligilactobacillus salivarius*, 85% of *Ligilactobacillus agilis*, and 58.8% of *Lactiplantibacillus plantarum* were classified as resistant to kanamycin, with the *aac(6)-Ie-aph(2)-Ia* gene detected only in two isolates. In six of 12 streptomycin-resistant strains, the *ant(6)-Ia* gene was identified, which usually coexisted with the *spw* gene. Three isolates with high neomycin MICs harbored the *ant(4′)-Ia* gene. In *Lactobacillus gallinarum* strain LMG 9435, characterized by streptomycin MIC value > 1024 µg/mL, a potential resistance-causing mutation in the *rpsL* gene (Lys56 → Arg) was detected. The results of the study indicate that some genera of *Lactobacillaceae*, in particular *L. salivarius* and *L. agilis*, exhibit natural resistance to aminoglycoside antibiotics, mainly kanamycin. Therefore, there is a need to update the EFSA guidelines on antimicrobial susceptibility testing of *Lactobacillaceae,* so that strains lacking resistance genes and/or chromosomal mutations are not considered to be resistant.

## 1. Introduction

*Lactobacillaceae* are gram-positive, aerotolerant or anaerobic, non-spore-forming bacilli or cocci. They are the most numerous group of lactic acid bacteria (LAB), which produce lactic acid as the major metabolic end-product of carbohydrate fermentation [1]. The *Lactobacillaceae* family currently includes 35 genera with >460 species. Of these, 19 are referred to as ‘candidatus’, meaning that they have provisional taxonomic names because they were characterized based on metagenome sequencing without obtaining pure cultures [2,3,4]. *Lactobacillaceae* inhabit nutrient-rich environments, such as the mucous membranes of humans and animals, milk and dairy products, plants, and fermented foods, and most species of this family are considered non-pathogenic. This makes them ideal candidates for probiotics, which are widely used in humans, livestock, and companion animals [5].

Probiotic strains should meet a number of criteria, some of which concern antimicrobial susceptibility. They cannot contain acquired resistance genes, especially those located on mobile genome elements, due to the risk of transferring them to other bacteria (e.g., in the host’s intestine), including opportunistic and pathogenic bacteria [6].

According to the EFSA Panel on Additives and Products or Substances used in Animal Feed (FEEDAP) guidelines published in 2018 [6], microorganisms used as feed additives or as production organisms in the European Union (EU) must be assessed for antimicrobial susceptibility based on both a phenotypic test and a whole-genome sequence analysis for the detection of possible resistance genes. In the phenotypic test for *Lactobacillaceae*, the MIC values of nine antimicrobial substances should be determined, including three aminoglycosides, i.e., kanamycin, gentamicin, and streptomycin [6]. However, details of the antimicrobial susceptibility testing (AST) have not been specified. Both the agar dilution method and the broth dilution method are accepted, and the AST ‘should be performed in accordance with internationally recognized standards such as the European Committee on Antibacterial Susceptibility Testing (EUCAST), the Clinical and Laboratory Standards Institute (CLSI), the ISO standard or similar’. FEEDAP only stipulates that the culture medium should enable the growth of the strains tested, and in the case of some LAB, LAB susceptibility medium (LSM) [7] can be used.

EFSA guidelines for determining the antimicrobial susceptibility of *Lactobacillaceae* are therefore imprecise, and the breakpoints are the same for different laboratory protocols. This approach is flawed because the MIC value depends on many factors, e.g., the composition of the culture medium, inoculum density, incubation time, temperature, and atmosphere [7,8,9,10,11,12,13]. In addition, although a new taxonomic division of the former genus *Lactobacillus* was published in 2020 [4], the EFSA breakpoints still refer to the old nomenclature and metabolic groups, i.e., *Lactobacillus* obligate heterofermentative (OHE), *Lactobacillus* obligate homofermentative (OHO), and *Lactobacillus* facultative heterofermentative (FHE). Separate cut-off points were set for the *Lactobacillus acidophilus* group (although EFSA does not specify which species should be included in this group), the species *Lactobacillus reuteri* (currently *Limosilactobacillus reuteri*), *Lactobacillus plantarum/pentosus* (currently *Lactiplantibacillus plantarum*), *Lactobacillus rhamnosus* (currently *Lacticaseibacillus rhamnosus*), and *L. casei/paracasei* (currently *Lacticaseibacillus casei/paracasei*), and the genera *Pediococcus* and *Leuconostoc.* It is quite surprising that for the closely related species *L. rhamnosus* and *L. casei*/*L. paracasei*, which are FHE, the FEEDAP Panel established different cut-off points. This causes some confusion regarding the categorization of other species of the *L. casei* phylogenetic group, i.e., *L. zeae*. Literature data indicate that EFSA guidelines need to be improved because the level of antimicrobial susceptibility of individual taxa belonging to a given metabolic group may vary [14]. Significant differences have been observed even between species belonging to the same genus, e.g., *L. delbrueckii* and *L. gasseri/L. paragasseri* [15]. Moreover, in the case of kanamycin and streptomycin, the EFSA’s cut-off points are in contradiction with several reports [14,16,17,18], including the findings of Mayrhofer et al. in 2010 [17], which are referred to by the FEEDAP [6]. In that study, many *Lactobacillaceae* strains were classified as resistant to kanamycin or streptomycin (using EFSA cut-off points) while lacking resistance genes.

## 2. Materials and Methods

### 2.1. Purpose and Scope of the Research

**Figure 1:**
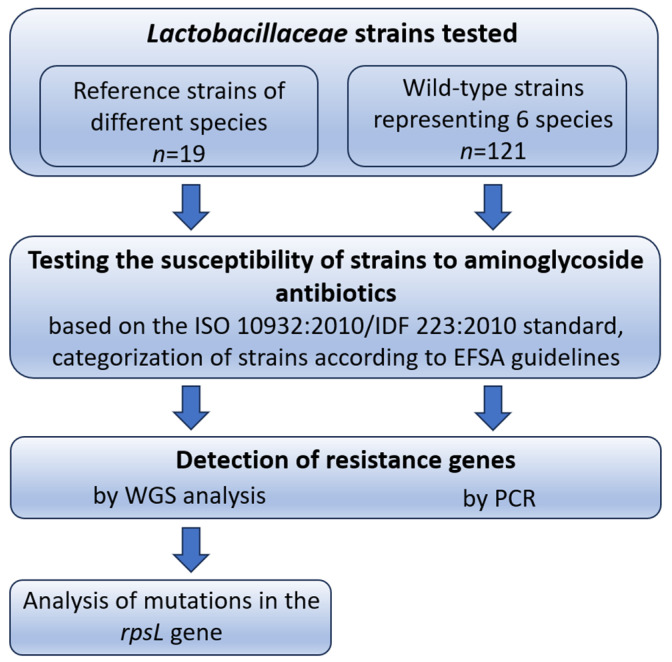
The study’s workflow.

The aim of the study was to determine the susceptibility of *Lactobacillaceae* strains (*n* = 140), both reference and wild-type, to aminoglycoside antibiotics (kanamycin, streptomycin, gentamicin, neomycin, spectinomycin) and to compare the results of the phenotypic test obtained based on EFSA breakpoints [6] with the results of genotypic analyses, i.e., detection of resistance genes and mutations in the *rpsL* gene. The study’s workflow is illustrated in Figure 1.

### 2.2. Strains and WGSs Used in the Study

The study used 19 reference strains and 121 wild-type strains of the *Lactobacillaceae* family (formerly the genus *Lactobacillus*) (Table 1). Wild-type strains were isolated from fecal samples from birds (chickens, turkeys, geese, pigeons) (*n* = 99), dogs (*n* = 12), and from commercial probiotic preparations (*n* = 6). Detailed information about the isolates and their sources of origin is provided in Table S1. The isolates were identified using MALDI-TOF mass spectrometry [18,19,20], and some were additionally identified based on the analysis of the 16S–23S rDNA regions [21] and the 16S rDNA gene [22].

The WGSs of the reference strains were downloaded from the GenBank database (Table 1). The WGSs of two randomly selected aminoglycoside-resistant wild-type isolates, i.e., *Lactiplantibacillus plantarum* G2Lp and *Ligilactobacillus salivarius* T25a, and one aminoglycoside-susceptible strain of *Lactobacillus crispatus* (T31e), were generated by nanopore sequencing according to protocol SQK-RBK114.96 with Flow Cell version R10.4 on a MinION device (FLO-MIN106D; Oxford Nanopore, Oxford, UK), using the super-accurate base-calling method in MinKNOW v22.12.7. Reads were trimmed and down-sampled to 200× coverage using Filtlong v0.20 and assembled into circular contigs using Flye v2.9.1 [23]. Genomes were polished using Medaka v1.11.0 and Homopolish v0.3.4 [24] and annotated using Prokka v1.14.5 [25].

### 2.3. Antimicrobial Susceptibility Testing

The antimicrobial susceptibility of the *Lactobacillaceae* strains was determined by the broth microdilution method using LSM containing 90% Iso-sensitest broth (Thermo Scientific, Basingstoke, UK) and 10% MRS (Biocorp, Warsaw, Poland) [7]. The antimicrobial agents included in the study were kanamycin (A&A Biotechnology, Gdynia, Poland), gentamicin, streptomycin, neomycin, and spectinomycin (Merck, Warsaw, Poland), although the latter was used only for reference strains. The analysis was based on the ISO 10932:2010/IDF 223:2010 standard [26]. Briefly, bacteria were suspended in 0.85% NaCl solution to obtain a turbidity of 1.0–1.1 according to the McFarland scale. The inocula were then diluted 1:500 in LSM broth. Stock solutions of antibiotics (20 mg/mL) were prepared in water (kanamycin and streptomycin) or in BRII buffer (16.73 g K_2_HPO_4_ and 0.523 g KH_2_PO_4_ dissolved in 1 L H_2_O, pH = 7.9) (gentamicin and neomycin), taking into account the product’s potency (given by the manufacturer and expressed in µg/1 mg of powder). Antibiotic dilutions were performed manually on a 96-well microplate in LSM. Finally, 50 µL of bacterial suspension was added to 50 µL of antibiotic solution [20]. For quality control, the *Lacticaseibacillus paracasei* ATCC 334 strain was used in each test repetition, in accordance with the ISO 10932:2010/IDF 223:2010 standard [26]. Plates were incubated at 37 °C in 5% CO_2_. MIC values (the lowest concentration of an antimicrobial agent at which no visible growth was observed) were read visually after 24 h [7] (two independent replicates for reference strains; no replicates for most field isolates) and after 48 h [26] (three independent replicates for reference strains; no replicates for most field isolates).

The categorization of strains into susceptible or resistant was based on the MIC cut-offs recommended by EFSA [6]. If different MIC values were obtained in subsequent test repetitions, the MIC with the highest value was finally taken into account. The categorization did not include neomycin and spectinomycin due to the lack of available guidelines. For strains of the species *L. acidophilus*, *Lactobacillus johnsonii*, *Lactobacillus paragasseri*, *Lactobacillus gallinarum*, *Lactobacillus amylovorus*, *Lactobacillus kitasatonis*, and *Lactobacillus crispatus*, EFSA breakpoints for the *L. acidophilus* group were used [17]. Strains of *Limosilactobacillus oris* and *Limosilactobacillus ingluviei* were assessed according to the criteria for OHE lactobacilli, and strains of *Ligilactobacillus salivarius* and *Ligilactobacillus agilis* according to the criteria for FHE lactobacilli [1,6].

### 2.4. Detection of Resistance Genes

For the *Lactobacillaceae* reference strains and three wild-type strains for which WGSs were obtained (G2Lp, T25a, T31e), resistance genes were detected based on WGS analysis using Resfinder 4.1 [27], Resistance Gene Identifier (RGI) ver. 6.0.3 [28], and ABRicate ver. 0.8 [29]. In the remaining field isolates (*n* = 118), aminoglycoside resistance genes were detected using PCR. The *aac(6)-Ie-aph(2)-Ia*, *ant(4′)-Ia, aph(2″)-Ib*, *aph(2″)-Ic* and *aph(2″)-Id* genes were detected by multiplex PCR 1 [30], and the multiplex PCR 2 developed by us earlier was used to detect the *ant(6)-Ia* (*aadE*), *spw*, and *aph(3′)-IIIa* genes [31]. Primer sequences, amplicon sizes, and annealing temperature are given in Table S2. Wild-type LAB strains containing resistance genes were used as positive controls (Table S3).

### 2.5. Analysis of Mutations in the rpsL Gene

To explain the mechanism of resistance of the reference *Lactobacillaceae* strains to streptomycin, the sequence of the *rpsL* gene encoding the 30S subunit ribosomal protein S12 was analyzed. Due to the particularly high streptomycin MIC value (>1024 µg/mL) of *L. gallinarum* strain LMG 9435, the analysis also included other isolates of this species (with unknown susceptibility to streptomycin) (*n* = 4) and strains of other species belonging to the *L. delbrueckii* phylogenetic group with a previously determined degree of susceptibility to streptomycin (*n* = 8) [14] (Table A1). DNA sequences of the *rpsL* gene and putative protein sequences obtained by translation using ORF Finder (https://www.ncbi.nlm.nih.gov/orffinder/, accessed on 26 January 2025) were aligned in MEGA XI ver. 11.0.13.

## 3. Results

### 3.1. Results of AST and Resistance Gene Detection

According to EFSA criteria [6], after 48 h of incubation in LSM, 36.8% (7/19) of reference *Lactobacillaceae* strains showed resistance to kanamycin, 26.3% (5/19) were resistant to streptomycin, and 5.3% (1/19) showed resistance to gentamicin. The range of neomycin and spectinomycin MICs ranged from 1 to 64 µg/mL and from 4 to 256 µg/mL, respectively. Despite the significant percentage of resistant strains, no genes conferring resistance to aminoglycosides were detected in any genome (in several genomes, only genes associated with resistance to other antimicrobials were detected) (Table 2 and Table S4).

**Figure 2:**
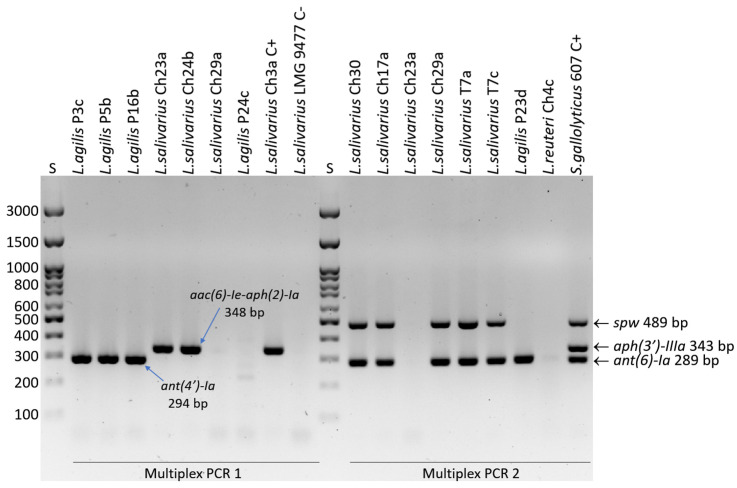
Electrophoretic separation of PCR products (amplicons of resistance genes) in a 2% agarose gel. S—DNA size standard; C+—positive control; C−—negative control.

In wild-type isolates, resistance to kanamycin was most frequently recorded (78/121; 64.5%), and less frequently to streptomycin (12/121; 9.9%) and gentamicin (3/121, 2.5%). Genes determining aminoglycoside resistance were detected in only 11 of 78 (14.1%) phenotypically resistant isolates (Table S1). As many as 93% of *L. salivarius* isolates, 85% of *L. agilis* isolates, and 58.8% of *L. plantarum* isolates, as well as 26.4–33.3% of *L. ingluviei* and *L. reuteri* isolates, were classified as resistant to kanamycin. However, no strains were found to contain the *aph(3′)-IIIa* gene, which usually confers resistance to kanamycin in bacteria, but two *L. salivarius* isolates with very high MICs for kanamycin (≥1024 µg/mL) and gentamicin (≥128 µg/mL) revealed *the aac(6)-Ie-aph(2)-Ia* gene (coding for bifunctional aminoglycoside acetyltransferase). Interestingly, one of the *aac(6)-Ie-aph(2)-Ia*-positive strains also had a very high MIC of streptomycin (>1024 µg/mL) (Table S1). Streptomycin and gentamicin resistance were recorded only in isolates of *L. salivarius* and *L. agilis*. In six of 12 isolates resistant to streptomycin and characterized by high MICs (≥512 µg/mL), the *ant(6)-Ia* (*aadE*) gene (encoding aminoglycoside nucleotidyltransferase) was detected, and in *L. salivarius* isolates it coexisted with the *spw* gene (encoding aminoglycoside nucleotidyltransferase of the ANT(9) family). In 3 *L. agilis* strains with high neomycin MICs (128–256 µg/mL), the presence of the *ant(4′)-Ia* gene (encoding aminoglycoside O-nucleotidyltransferase) was confirmed (Table 3 and Table S1, Figure 2).

For all reference and wild-type strains, MIC values could only be read after 24 h of incubation, although *L. acidophilus* strain ATCC 4356 and some *L. reuteri* isolates displayed poor growth. The most intensive growth in LSM medium, assessed visually, was observed in isolates of *L. salivarius* and *L. agilis*. The MICs were usually one order of magnitude lower than the reading after 48 h, which resulted in a slightly lower percentage of resistant strains (Table 2, Tables S1 and S2).

### 3.2. Mutations in the rpsL Gene

Mutations in the *rpsL* gene encoding the S12 protein, which is part of the small ribosomal subunit, were detected in two of the 22 strains tested, i.e., *L. gallinarum* strain LMG 9435 and *L. amylovorus* LMG 9496 (Table 2). The mutation was recorded in *L. gallinarum* LMG 9435 at position 167 (AAG → AGG), translating into a Lys56 → Arg substitution, which most likely determined the high streptomycin MIC value of > 1024 µg/mL. At the same time, no cross-resistance to other aminoglycoside antibiotics has been reported. By analyzing the *rpsL* sequences of other *L. gallinarum* strains (*n* = 4) (of unknown susceptibility to streptomycin), we ruled out the possibility that the mutation at this position was a constant feature of strains of this species. Additionally, based on AST results published by Campedelli et al. [14], we showed that the mutation at position 167 does not occur in streptomycin-susceptible strains (of various species) of the *L. delbrueckii* phylogenetic group. However, the same aa substitution (Lys56 → Arg) was noted in the streptomycin-resistant *L. gigeriorum* strain DSM 23908 (Table A1). The mutation noted in *L. amylovorus* strain LMG 9496 at position 303 (AAG → ACG), which translates into a substitution (Lys101 → Thr), does not seem to affect the susceptibility of this strain to streptomycin (MIC 16–32 µg/mL) (Table A1).

## 4. Discussion

In this study, in an antimicrobial susceptibility test based on the ISO 10932:2010/IDF 223:2010 standard [23] and using the EFSA/FEEDAP Panel cut-off points [6], 52.6% (10/19) of reference *Lactobacillaceae* strains and 64.5% (78/121) of wild-type strains were classified as resistant to aminoglycosides, most of them lacking resistance genes. Resistance to kanamycin was most frequently noted, less frequently to streptomycin and gentamicin. Our observations are consistent with results reported by several other authors who assessed the antimicrobial susceptibility of *Lactobacillaceae* according to the same protocol.

The widespread occurrence of kanamycin resistance (61%) among reference *Lactobacillaceae* strains of various species (former genus *Lactobacillus*) has also been noted by Campedelli et al. [14], who detected (by whole-genome analysis) genes that could determine resistance to this antibiotic, i.e., *aac(3)* and *aph(3)*, in only 11% of the phenotypically resistant strains. The same study also showed a significant percentage of strains to be resistant to streptomycin (27%) and gentamicin (14.2%), while the *ant(6)* and *ant(9)* resistance genes were identified in only in a few strains [14]. Mayrhofer et al. [17] reported resistance to kanamycin (MIC = 128 µg/mL) and streptomycin in 23.8% (24/101) and 8.9% (9/101) of strains of the *L. acidophilus* phylogenetic group, respectively, and did not detect aminoglycoside resistance genes in any strain. Results consistent with ours were also recently demonstrated by scientists from Denmark [15], who examined a total of 170 strains from the *Lactobacillaceae* family (from the Chr. Hansen’s Culture collection). According to the EFSA cut-off points, kanamycin resistance was demonstrated by 92% of *L. salivarius* strains, 11% of *L. delbrueckii* strains, and 13% of *L. gasseri*/*L. paragasseri* strains. Streptomycin resistance was recorded for 33% of *L. salivarius* strains, 60% of *L. sakei* strains, 4% of *L. delbrueckii* strains, and 6% of *L. gasseri*/*L. paragasseri* strains. No strain exceeded the FEEDAP Panel threshold values for gentamicin, and WGS analysis showed that none of the strains contained genes determining resistance to aminoglycosides [15]. In another study conducted on 65 LAB strains, including 57 from the former genus *Lactobacillus*, kanamycin resistance was recorded in 32.3% (21/65) of the strains, streptomycin-resistant strains accounted for 21.4% (12/56), and gentamicin-resistant for 3.1% (2/65). The *aph(3)-IIIa* gene was detected in only four of 21 (19%) kanamycin-resistant strains, and the *str(A)*/*str(B)* gene was detected in just one of 12 streptomycin-resistant strains (and in one phenotypically susceptible strain) [16]. In our previous paper, we showed widespread resistance of *Lactobacillaceae* isolates from pigeons to kanamycin (89%) and streptomycin (63%), and 14% of isolates were resistant to gentamicin [18]. However, it should be noted that the antimicrobial susceptibility test was performed in Iso-sensitest medium containing 25% MRS (not 10% as indicated in the ISO 10932:2010/IDF 223:2010 standard), which may have affected the MIC value. A high frequency of resistance to streptomycin has also been recorded in *Lactobacillaceae* strains (former genus *Lactobacillus*) from chickens (31%) and turkeys (31%), including *L. agilis* (33–100% of strains showed resistance to streptomycin), *L. crispatus* (17–23%), *L. salivarius* (50–52%), *L. saerimneri* (100%), and *L. johnsonii* (14%). However, the presence of the *ant(6)-Ia* gene was confirmed in only a few phenotypically resistant *L. salivarius* strains [19,20]. Danielsen and Wind [32], who used E-tests and MRS medium to assess the antimicrobial susceptibility of *Lactobacillaceae* (former genus *Lactobacillus*), reported high kanamycin and streptomycin MIC values (≥128 µg/mL) for 86% and 61% of isolates tested, respectively.

The results of this study, as well as the other studies cited above, suggest that the high frequency of kanamycin resistance in the examined species of the genus *Ligilactobacillus* and *Limosilactobacillus* is mainly due to intrinsic resistance, which is most likely linked to the lack of cytochrome-mediated drug transport [33]. The theory of intrinsic resistance is also supported by the unimodal distribution of kanamycin and streptomycin MIC values in *Lactobacillaceae*, including *L. salivarius* and *L. agilis*, also observed by other authors [15,18,32]. According to CLSI (document M100-Ed34) [34], intrinsic resistance is defined as ‘inherent or innate (not acquired) antimicrobial resistance, which is reflected in wild-type antimicrobial patterns of all or almost all representatives of a species’. Furthermore, the CLSI states that ‘intrinsic resistance is so common that susceptibility testing is unnecessary’. Therefore, it must be clearly determined which, if any, species of *Lactobacillaceae* are naturally resistant to aminoglycoside antibiotics, so that individual antimicrobial substances from this group can be excluded from laboratory tests. At the same time, it should be noted that EFSA does not rule out the use of naturally resistant strains as microbial feed additives. The guidelines state that ‘detection of the MIC above the cut-off values proposed by the FEEDAP Panel for one or more antimicrobials requires further investigation using genomic data to determine the nature of the resistance’, but ‘if no known AMR gene is identified that can be linked to the phenotype, no further studies are required’ [6].

*Lactobacillaceae* resistance to aminoglycosides may also be caused by mutations in chromosomal genes, e.g., those encoding ribosomal proteins, as we have shown in the case of streptomycin-resistant *L. gallinarum* strain LMG 9435. The mutation at position 167 (AAG → AGG) of the *rpsL* gene, translating into a Lys56 → Arg substitution, has previously been identified in streptomycin-resistant *L. plantarum* [35] and *L. rhamnosus* [36] strains. In the latter species, a mutation leading to a substitution for lysine was also recorded at position 303 [36]. In other taxonomic groups of bacteria, mutations determining resistance to streptomycin have been identified at other positions of the *rpsL* gene, i.e., at position 129 (Lys43 → Arg/Thr/Asn)) in *Bifidobacterium bifidum* [37], *Yersinia pestis* [38], and *Pectobacterium carotovorum* [39]; at position 128 (Lys42 → Thr) and 263 (Lys86 → Arg) in *E. coli* [40] and in *Mycobacterium tuberculosis* [41]. Interestingly, in almost all cases, streptomycin resistance was associated with a change from lysine to arginine or lysine to threonine. In agreement with our results are the findings of Bernard et al. [39] that streptomycin-resistant *Pectobacterium carotovorum* subsp. *carotovorum* (formerly *Erwinia carotovora* subsp. *carotovora*) strains with a mutation in the *rpsL* gene did not show cross-resistance to spectinomycin, kanamycin, or gentamicin.

We have demonstrated that the same cut-off points should not be used for different susceptibility testing protocols, as incubation time affects the MIC value, often leading to a change in the categorization of a strain from susceptible (reading after 24 h of incubation) to resistant (reading after 48 h). Similar conclusions have been reached by other authors analyzing the effect of incubation time and inoculum density on the antimicrobial susceptibility of LAB [8].

It should also be considered whether AST requires incubation of the microplate for as long as 48 h, given that many *Lactobacillaceae* species, especially *L. salivarius* (based on visual assessment), grow very well in LSM broth after 24 h. Longer incubation substantially decreases the pH of the medium due to lactic acid production, which may affect the activity of antimicrobial substances as well as the growth rate and viability of bacteria [42,43]. In addition, some antimicrobial substances, including neomycin, have limited stability in the culture medium [44]. Another possible undesirable effect of 48 h incubation may be partial evaporation of the medium from the microplate wells, which may also affect the MIC value.

Neomycin was included in this study because, unlike kanamycin, it is often used in poultry, pig, and cattle farming. Natural resistance to neomycin would certainly be a desirable feature to take into account when selecting probiotic strains for these groups of animals. We therefore believe that it would be worth extending the EFSA guidelines to include neomycin. Establishing cut-off points for this antibiotic, however, requires testing on a large number of *Lactobacillaceae* strains.

## 5. Conclusions

Our study shows that categorization of *Lactobacillaceae* strains (representing the former genus *Lactobacillus*) according to the cut-off points established by the EFSA FEEDAP Panel in 2018 [6] and the use of the antimicrobial susceptibility protocol according to ISO 10932:2010/IDF 223:2010 [26] can lead to the recognition of strains that do not contain resistance genes as resistant, and thus eliminate them from further studies on the selection of probiotic strains. It is true that in addition to the phenotypic test, EFSA also requires WGS analyses to detect resistance genes. However, in the standard laboratory procedure, the phenotypic test is performed first, and only those strains that do not exceed the cut-off points are referred for WGS (due to the high cost of genomic sequencing).

Our results, as well as other reports mentioned above, have shown that the cut-off points established by EFSA for aminoglycoside antibiotics, especially kanamycin, should be re-examined, defined at the genus and/or species level, and adapted to the given protocol of AST. It would also be appropriate to expand the guidelines to include neomycin, which is commonly used in animal treatment. The data presented in this paper may be useful for revising the current EFSA guidelines on the AST of *Lactobacillaceae*.

## Figures and Tables

**Table 1:** Reference and wild-type *Lactobacillaceae* strains used in the study.

Reference Strain	Other Culture Collection Numbers	WGS [GenBank Acc. No.]
*Ligilactobacilluis salivarius* LMG 9476	DSM 20554; ATCC 11742	GCA_002079585.1
*Ligilactobacillus salivarius* LMG 9477	DSM 20555; ATCC 11741; JCM 1231	GCA_001435955.1
*Ligilactobacillus agilis* LMG 9186	DSM 20509; LMG 9186	GCA_001436215.1
*Lactiplantibacillus plantarum* ATCC 8014	DSM 20205; JCM 1057; LMG 1284	GCA_002631775.1
*Lacticaseibacillus rhamnosus* ATCC 7469	DSM 20021; JCM 1136	GCA_001435405.1
*Lacticaseibacillus casei* ATCC 393	DSM 20011; JCM 1134	GCA_000829055.1
*Lacticaseibacillus zeae* LMG 17315	DSM 20178; JCM 11302; ATCC 15820	GCA_001433745.1
*Limosilactobacillus reuteri* subsp. *reuteri* LMG 9213	ATCC 23272; JCM 1112; DSM 20016	GCA_000010005.1
*Limosilactobacillus reuteri* LMG 18238	ATCC 55148	ERR3330657
*Limosilactobacillus ingluviei* LMG 20380	JCM 12531; DSM 15946; CCUG 45722	GCA_001435775.1
*Limosilactibacillus ingluviei* LMG 22056	DSM 14792; JCM 11425	GCA_001437235.1
*Limosilactibacillus oris* LMG 9848	DSM 4864; ATCC 49062	GCA_001434465.1
*Lactobacillus paragasseri* LMG 13134	LMG 11444; JCM 5344; ATCC 9857	GCA_003307295.1
*Lactobacillus acidophilus* ATCC 4356	DSM 20079; JCM 1132; NCIB 8690	GCA_034298135.1
*Lactobacillus gallinarum* LMG 9435	JCM 2011; ATCC 33199; DSM 10532	GCA_001434975.1
*Lactobacillus kitasatonis* LMG 23133	LMG 22685; JCM 1039, DSM 16761	GCA_000615285.1
*Lactobacillus amylovorus* LMG 9496	JCM 1126; ATCC 33620; DSM 20531	GCA_002706375.1
*Lactobacillus johnsonii* LMG 9436	ATCC 33200; JCM 2012; DSM 10533	GCA_001433975.1
*Lactobacillus crispatus* LMG 9479	ATCC 33820; DSM 20584; JCM 1185	GCA_018987235.1
Wild-type Strains		
*Ligilactobacillus salivarius* (*n* = 44) *Ligilactobacillus agilis* (*n* = 20) *Lactiplantibacillus plantarum* (*n* = 17) *Limosilactobacillus ingluviei* (*n* = 20) *Limosilactobacillus reuteri* (*n* = 16) *Lactobacillus crispatus* (*n* = 4)		

**Table 2:** MIC values (µg/mL) obtained in the antimicrobial susceptibility test.

Antibiotic→	Kanamycin		Streptomycin		Spectinomycin	Gentamicin		Neomycin		Resistance Genes Detected
EFSA Cut-Offs→	R > 64 µg/mL		R > 64 µg/mL or R > 32 µg/mL ^A^ or R > 16 µg/mL ^B^		NA	R > 16 µg/mL or R > 8 µg/mL ^C^ or R > 32 µg/mL ^D^		NA		
Strain	24 h	48 h	24 h	48 h	48 h	24 h	48 h	24 h	48 h	
*L. salivarius* LMG 9476	64–128	128–512	32	32–64	32	4–8	8	8–16	8–16	
*L. salivarius* LMG 9477	64–128	128–256	32	32–64	64	4–8	4–8	8–16	8–16	
*L. agilis* LMG 9186	512	512	128	128–256	256	16–32	16–32	16–32	32–64	
*L. reuteri* subsp. *reuteri* LMG 9213	16–32	16–32	8–16	8–32	128	0.5–1	0.5–2	1–4	2–4	
*L. reuteri* LMG 18238	64–256	128–256	16–64	32–64	128	4	4–8	16	16	
*L. oris* LMG 9848	16–32	32–64	8–16	16–64	128	0.5–1	1	2–4	2–4	
*L. ingluviei* LMG 20380	32	64	64–128	64–128	256	2–4	2–4	8	8	*tetW*, *tetM*, *tetL*, *ermB*
*L. ingluviei* LMG 22056	64–128	128–256	32	32–64	256	2	4–8	16	16	*tetW*, *lnuC*
*L. plantarum* ATCC 8014	4–8	16	4–8	4–16	128	0.25	0.25	1–2	1–4	
*L. rhamnosus* ATCC 7469	16	32–64	4	8–16	64	1–2	2–4	2–8	4–16	
*L. casei* ATCC 393	16	32–64	8	16	32	1–2	2	4	8	
*L. zeae* LMG 17315	32–64	64–128	16–32	32	64	4	4	8–16	8–32	
*L. johnsonii* LMG 9436	32–64	64	8–8	8–16	32	4	4–8	8–32	8–32	
*L. paragasseri* LMG 13134	64	64	2–4	4–8	8	2–4	2–4	16–32	32–64	
*L. acidophilus* ATCC 4356	4	4	8	4–16	16	1–2	1–2	1–4	4	
*L. gallinarum* LMG 9435	16	16	>1024	>1024	16	0.25	0.25–0.5	4	8	*tetW*, *rpsL*_Arg56_
*L. kitasatonis* LMG 23133	8–16	8–16	1–2	2–8	16	2	2–4	32	32–64	
*L. amylovorus* LMG 9496	8–16	16	8–16	8–32	4	1	2	8	16	*rpsL*_Thr101_
*L. crispatus* LMG 9479	128	128	16	16–32	32	4–8	8	16–32	32	
Total resistant	6 [31.6%]	7 [36.8%]	3 [15.8%]	5 [26.3%]	NA	1 [5.3%]	1 [5.3%]	NA	NA	

Gray-highlighted values indicate resistance; ^A^—applies to *L. rhamnosus*; ^B^—applies to species from the *L. acidophilus* group; ^C^—applies to *L. reuteri*; ^D^—applies to *L. casei*/*paracasei* and *L. zeae*; NA—not applicable.

**Table 3:** Distribution of MIC values of aminoglycoside antibiotics in wild-type *Lactobacillaceae* strains.

MIC Value→ [µg/mL]	≤0.25	0.5	1	2	4	8	16	32	64	128	256	512	≥1024	No. [%] of Resistant
Kanamycin														
*L. salivarius* (*n* = 44)									3	20	19		2 ^bif^	41 [93.2]
*L. agilis* (*n* = 20)									3	10	4	2 *^ant(4)Ia^*	1	17 [85.0]
*L. plantarum* (*n* = 17)							3	2	2	8	2			10 [58.8]
*L. ingluviei* (*n* = 18)							1	2	9	5	1			6 [33.3]
*L. reuteri* (*n* = 15)					1	1	5	4		4				4 [26.7]
*L. crispatus* (*n* = 4)								1	3					0
Streptomycin														
*L. salivarius* (*n* = 44)							3	13	19	3		2 *^spw^* ^*aadE*^	4 *^spw^*^(3)^ ^*aadE*(3)^	9 [20.4]
*L. agilis* (*n* = 20)						1	3	5	8	2			1 *^aadE^*	3 [15.0]
*L. plantarum* (*n* = 17)						1	2	7	6	1				NA
*L. ingluviei* (*n* = 20)							2	8	10					0
*L. reuteri* (*n* = 16)					3	6	2	4	1					0
*L. crispatus* (*n* = 4)				2	1		1							0
Gentamicin														
*L. salivarius* (*n* = 44)			1	5	14	17	5			1 ^bif^			1 ^bif^	2 [4.5]
*L. agilis* (*n* = 20)			2	3	9	4	1	1						1 [5.0]
*L. plantarum* (*n* = 17)		2	2	6	1	6								0
*L. ingluviei* (*n* = 20)		1	2	12	3	1	1							0
*L. reuteri* (*n* = 16)	3	5	3	3	2									0
*L. crispatus* (*n* = 4)				2	2									0
Neomycin														
*L. salivarius* (*n* = 41)				4	11	20	5	1						NA
*L. agilis* (*n* = 20)				1	5	3	6	1		3 *^ant(4)Ia^*^(2)^	1 *^ant(4)Ia^*			NA
*L. plantarum* (*n* = 11)			1	3	1	3	2	1						NA
*L. ingluviei* (*n* = 20)			1	7	8	3	1							NA
*L. reuteri* (*n* = 13)	2	2	4	3		1	1							NA
*L. crispatus* (*n* = 4)						1	2	1						NA

Grey-highlighted values (µg/mL) indicate resistance; NA—not applicable; bif—*aac(6)-Ie-aph(2)-Ia gene*; the number of strains carrying the gene in question is given in brackets after the name of gene; the absence of any number following the name of gene means that all isolates contain the gene

## Data Availability

The WGSs reported in this paper have been deposited in the NCBI GenBank database under the following accession numbers: CP168064-CP168068 (GCA_041531555.1)—strain G2Lp, CP180626-CP180627 (GCA_047782765.1)—strain T31e, CP183830-CP183833 (GCA_048572735.1)—strain T25a.

## Supplementary Materials

The following supporting information can be downloaded at https://www.mdpi.com/article/10.3390/life15050732/s1: Table S1: Results of antimicrobial susceptibility testing (MIC values) and detection of resistance genes in *Lactobacillaceae* isolates; Table S2: Primers used for detection of aminoglycoside resistance genes; Table S3: Bacterial strains used as positive control during detection of resistance genes by PCR; Table S4: MIC values (µg/mL) obtained in the antimicrobial susceptibility test for reference *Lactobacillaceae* strains.

## Abbreviations

The following abbreviations are used in this manuscript:

AST	Antimicrobial susceptibility testing
CLSI	Clinical and Laboratory Standards Institute
EFSA	European Food Safety Authority
FEEDAP	EFSA Panel on Additives and Products or Substances used in Animal Feed
IDF	International Dairy Federation
ISO	International Organization for Standardization
LAB	Lactic acid bacteria
LSM	LAB susceptibility test medium
MRS	De Man-Rogosa-Sharpe
WGS	Whole genome sequence

## Appendix A

**Table A1:** Results of mutation detection in the *rpsL* gene encoding ribosomal protein S12 in strains of the *L. delbrueckii* phylogenetic group.

Strain	Metabolic Group	Phylogenetic Group	GenBank Acc. No.	Susceptibility to STR	MIC Value	Mutations in *rpsL* Gene	Aminoglycoside RGs
*L. gallinarum* LMG 9435	OHO	*L. delbrueckii*	GCA_001434975.1	R	>1024	Lys56 → Arg	No
*L. gallinarum* An153	OHO	*L. delbrueckii*	GCA_002160635.1	ND	ND		No
*L. gallinarum* An101	OHO	*L. delbrueckii*	GCA_002161165.1	ND	ND		No
*L. gallinarum* J07	OHO	*L. delbrueckii*	GCA_947381795.1	ND	ND		No
*L. gallinarum* Chicken_20_mag_156	OHO	*L. delbrueckii*	GCA_904419645.1	ND	ND		No
*L. acidophilus* ATCC 4356	OHO	*L. delbrueckii*	GCA_034298135.1	S	4–16		No
*L. amylovorus* LMG 9496	OHO	*L. delbrueckii*	GCA_002706375.1	S/R	8–32	Lys101 → Thr	No
*L. crispatus* LMG 9479	OHO	*L. delbrueckii*	GCA_018987235.1	S/R	16–32		No
*L. crisptus* T31e	OHO	*L. delbrueckii*	GCA_047782765.1	S	8–16		No
*L. amylolyticus* DSM 11664	OHO	*L. delbrueckii*	GCA_004354545.1	S [14]	UN		No
*L. gigeriorum* DSM 23908	OHO	*L. delbrueckii*	GCA_001436575.1	R [14]	UN	Lys56 → Arg	No
*L. delbrueckii* subsp. *jakobsenii* DSM 26046	OHO	*L. delbrueckii*	GCA_001888925.1	R [14]	UN		No
*L. delbruckii* subsp. *delbrueckii* DSM 20074	OHO	*L. delbrueckii*	GCA_001908495.1	S [14]	UN		No
*L. helveticus* LMG 22464	OHO	*L. delbrueckii*	GCA_001437535.1	S [14]	UN		No
*L. taiwanensis* DSM 21401	OHO	*L. delbrueckii*	GCA_001436695.1	S [14]	UN		No
*L. pasteurii* DSM 23907	FHE	*L. delbrueckii*	GCA_004354755.1	S [14]	UN		No
*L. apis* LMG 26964	FHE	*L. delbrueckii*	GCA_002837055.1	R [14]	UN		No

OHO—obligate homofermentative; FHE—facultative heterofermentative; R—resistant; S—susceptible; ND—not determined; UN—unknown; RGs—resistance genes.

## References

[1] Sun Z., Yu J., Dan T., Zhang W., Zhang H., Zhang H., Cai Y. (2014). Phylogenesis and evolution of lactic acid bacteria. Lactic Acid Bacteria.

[2] Gilroy R., Ravi A., Getino M., Pursley I., Horton D.L., Alikhan N.F., Baker D., Gharbi K., Hall N., Watson M. (2021). Extensive microbial diversity within the chicken gut microbiome revealed by metagenomics and culture. PeerJ.

[3] NCBI Database. https://www.ncbi.nlm.nih.gov/taxonomy.

[4] Zheng J., Wittouck S., Salvetti E., Franz C.M.A.P., Harris H.M.B., Mattarelli P., O’Toole P.W., Pot B., Vandamme P., Walter J. (2020). taxonomic note on the genus *Lactobacillus*: Description of 23 novel genera, emended description of the genus *Lactobacillus* Beijerinck 1901, and union of Lactobacillaceae and Leuconostocaceae. Int. J. Syst. Evol. Microbiol..

[5] Bhogoju S., Nahashon S. (2022). Recent Advances in Probiotic Application in Animal Health and Nutrition: A Review. Agriculture.

[6] EFSA (2018). Guidance on the characterisation of microorganisms used as feed additives or as production organisms. EFSA J..

[7] Klare I., Konstabel C., Müller-Bertling S., Reissbrodt R., Huys G., Vancanneyt M., Swings J., Goossens H., Witte W. (2005). Evaluation of new broth media for microdilution antibiotic susceptibility testing of Lactobacilli, Pediococci, Lactococci, and Bifidobacteria. Appl. Environ. Microbiol..

[8] Egervärn M., Lindmark H., Roos S., Huys G., Lindgren S. (2007). Effects of inoculum size and incubation time on broth microdilution susceptibility testing of lactic acid bacteria. Antimicrob. Agents Chemother..

[9] Mayrhofer S., Zitz U., Birru F.H., Gollan D., Gołoś A.K., Kneifel W., Domig K.J. (2014). Comparison of the CLSI guideline and ISO/IDF standard for antimicrobial susceptibility testing of Lactobacilli. Microb. Drug Resist..

[10] Łepecka A., Szymański P., Rutkowska S., Iwanowska K., Kołożyn-Krajewska D. (2021). The Influence of Environmental Conditions on the Antagonistic Activity of Lactic Acid Bacteria Isolated from Fermented Meat Products. Foods.

[11] Tscherne A., Mantel E., Boskani T., Budniak S., Elschner M., Fasanella A., Feruglio S.L., Galante D., Giske C.G., Grunow R. (2022). Adaptation of *Brucella melitensis* Antimicrobial Susceptibility Testing to the ISO 20776 Standard and Validation of the Method. Microorganisms.

[12] Sabath L.D. (1977). Chemical and physical factors influencing methicillin resistance of *Staphylococcus aureus* and *Staphylococcus epidermidis*. J. Antimicrob. Chemother..

[13] Mendonça A.A., de Morais M.A., Cabrera M.Z. (2019). Cysteine induces resistance of lactobacilli to erythromycin and azithromycin. Int. J. Antimicrob. Agents..

[14] Campedelli I., Mathur H., Salvetti E., Clarke S., Rea M.C., Torriani S., Ross R.P., Hill C., O’Toole P.W. (2018). Genus-Wide Assessment of Antibiotic Resistance in *Lactobacillus* spp.. Appl. Environ. Microbiol..

[15] Nøhr-Meldgaard K., Struve C., Ingmer H., Koza A., Al-Nakeeb K., Agersø Y. (2023). Antimicrobial susceptibility testing and tentative epidemiological cut-off values for Lactobacillaceae family species intended for ingestion. Front. Antibiot..

[16] Stefańska I., Kwiecień E., Jóźwiak-Piasecka K., Garbowska M., Binek M., Rzewuska M. (2021). Antimicrobial Susceptibility of Lactic Acid Bacteria Strains of Potential Use as Feed Additives—The Basic Safety and Usefulness Criterion. Front. Vet. Sci..

[17] Mayrhofer S., van Hoek A.H., Mair C., Huys G., Aarts H.J., Kneifel W., Domig K.J. (2010). Antibiotic susceptibility of members of the *Lactobacillus* acidophilus group using broth microdilution and molecular identification of their resistance determinants. Int. J. Food Microbiol..

[18] Dec M., Stępień-Pyśniak D., Nowaczek A., Puchalski A., Urban-Chmiel R. (2020). Phenotypic and genotypic antimicrobial resistance profiles of fecal lactobacilli from domesticated pigeons in Poland. Anaerobe..

[19] Dec M., Nowaczek A., Stępień-Pyśniak D., Wawrzykowski J., Urban-Chmiel R. (2018). Identification and antibiotic susceptibility of lactobacilli isolated from turkeys. BMC Microbiol..

[20] Dec M., Urban-Chmiel R., Stępień-Pyśniak D., Wernicki A. (2017). Assessment of antibiotic susceptibility in *Lactobacillus* isolates from chickens. Gut Pathog..

[21] Dec M., Urban-Chmiel R., Gnat S., Puchalski A., Wernicki A. (2014). Identification of *Lactobacillus* strains of goose origin using MALDI-TOF mass spectrometry and 16S-23S rDNA intergenic spacer PCR analysis. Res. Microbiol..

[22] Dec M., Puchalski A., Urban-Chmiel R., Wernicki A. (2016). 16S-ARDRA and MALDI-TOF mass spectrometry as tools for identification of *Lactobacillus* bacteria isolated from poultry. BMC Microbiol..

[23] Kolmogorov M., Yuan J., Lin Y., Pevzner P.A. (2019). Assembly of long, error-prone reads using repeat graphs. Nat. Biotechnol..

[24] Huang Y.T., Liu P.Y., Shih P.W. (2021). Homopolish: A method for the removal of systematic errors in nanopore sequencing by homologous polishing. Genome Biol..

[25] Seemann T. (2014). Prokka: Rapid prokaryotic genome annotation. Bioinformatics.

[26] (2010). Milk and Milk Products—Determination of the Minimal Inhibitory Concentration (Mic) of Antibiotics Applicable to Bifidobacteria and Non-Enterococcal Lactic Acid Bacteria (LAB).

[27] Bortolaia V., Kaas R.S., Ruppe E., Roberts M.C., Schwarz S., Cattoir V., Philippon A., Allesoe R.L., Rebelo A.R., Florensa A.F. (2020). ResFinder 4.0 for predictions of phenotypes from genotypes. J. Antimicrob. Chemother..

[28] Alcock B.P., Raphenya A.R., Lau T.T.Y., Tsang K.K., Bouchard M., Edalatmand A., Huynh W., Nguyen A.V., Cheng A.A., Liu S. (2020). CARD 2020: Antibiotic resistome surveillance with the comprehensive antibiotic resistance database. Nucleic Acids Res..

[29] Seemann T. Abricate, Github. https://github.com/tseemann/abricate.

[30] Vakulenko S.B., Donabedian S.M., Voskresenskiy A.M., Zervos M.J., Lerner S.A., Chow J.W. (2003). Multiplex PCR for detection of aminoglycoside resistance genes in enterococci. Antimicrob. Agents Chemother..

[31] Dec M., Nowak T., Webster J., Wódz K. (2024). Serotypes, Antimicrobial Susceptibility, and Potential Mechanisms of Resistance Gene Transfer in *Erysipelothrix rhusiopathiae* Strains from Waterfowl in Poland. Int. J. Mol. Sci..

[32] Danielsen M., Wind A. (2003). Susceptibility of *Lactobacillus* spp. to antimicrobial agents. Int. J. Food Microbiol..

[33] Hummel A.S., Hertel C., Holzapfel W.H., Franz C.M. (2007). Antibiotic resistances of starter and probiotic strains of lactic acid bacteria. Appl. Environ. Microbiol..

[34] (2024). Performance Standards for Antimicrobial Susceptibility Testing CLSI M100 Includes Updated Tables for the Clinical and Laboratory Standards Institute Antimicrobial Susceptibility Testing Standards CLSI M02, M07, and M11. A CLSI Supplement for Global Application.

[35] Zhang F., Gao J., Wang B., Huo D., Wang Z., Zhang J., Shao Y. (2018). Whole-genome sequencing reveals the mechanisms for evolution of streptomycin resistance in *Lactobacillus* plantarum. J. Dairy Sci..

[36] Namai F., Nishiyama K., Kitazawa H., Shimosato T. (2024). Introduction of Spontaneous Mutations Using Streptomycin as a Method for Lactic Acid Bacteria Breeding. Methods Mol. Biol..

[37] Sirichoat A., Flórez A.B., Vázquez L., Buppasiri P., Panya M., Lulitanond V., Mayo B. (2020). Antibiotic Susceptibility Profiles of Lactic Acid Bacteria from the Human Vagina and Genetic Basis of Acquired Resistances. Int. J. Mol. Sci..

[38] Dai R., He J., Zha X., Wang Y., Zhang X., Gao H., Yang X., Li J., Xin Y., Wang Y. (2021). A novel mechanism of streptomycin resistance in Yersinia pestis: Mutation in the rpsL gene. PLoS Negl. Trop. Dis..

[39] Barnard A.M.L., Simpson N.J.L., Lilley K.S., Salmond G.P.C. (2010). Mutations in rpsL that confer streptomycin resistance show pleiotropic effects on virulence and the production of a carbapenem antibiotic in *Erwinia carotovora*. Microbiology.

[40] Pelchovich G., Schreiber R., Zhuravlev A., Gophna U. (2013). The contribution of common rpsL mutations in *Escherichia coli* to sensitivity to ribosome targeting antibiotics. Int. J. Med. Microbiol..

[41] Khosravi A.D., Etemad N., Hashemzadeh M., Khandan Dezfuli S., Goodarzi H. (2017). Frequency of rrs and rpsL mutations in streptomycin-resistant *Mycobacterium tuberculosis* isolates from Iranian patients. J. Glob. Antimicrob. Resist..

[42] Vera-Peña M.Y., Rodriguez Rodriguez W.L. (2020). Effect of pH on the growth of three lactic acid bacteria strains isolated from sour cream. Univ. Sci..

[43] Giraud E., Lelong B., Raimbault M. (1991). Influence of pH and initial lactate concentration on the growth of *Lactobacillus plantarum*. Appl. Microbiol. Biotechnol..

[44] Kerek Á., Ecsedi B.G., Szabó Á., Szimrók Z., Paliczné Kustán B., Jerzsele Á., Nagy G. (2024). Stability Studies of the Dilution Series of Different Antibiotic Stock Solutions in Culture Medium Incubated at 37 °C. Antibiotics.

